# Family perspectives on independent living for adults with cerebral palsy: barriers, support needs, and quality of life

**DOI:** 10.3389/fpsyt.2026.1785081

**Published:** 2026-03-11

**Authors:** Virginia Aguayo, Laura Esteban, Laura García-Domínguez, Miguel A. Verdugo

**Affiliations:** University Institute for Community Integration, Department of Personality, Evaluation and Psychological Treatment, University of Salamanca, Salamanca, Spain

**Keywords:** cerebral palsy, family caregivers, independent living, social participation, support systems

## Abstract

**Introduction:**

Independent living is recognized as a fundamental right under Article 19 of the Convention on the Rights of Persons with Disabilities. However, for many adults with cerebral palsy (CP) exercising this right remains challenging. Many adults with CP continue to rely on family caregivers or institutional arrangements, with consequences for autonomy and quality of life. Clinical complexity and structural barriers place families in a central position in shaping adult life trajectories. This study aimed to explore family caregivers’ understandings of independent living, the barriers they encounter, and the supports they identify as necessary to promote quality of life for adults with CP and their families.

**Methods:**

An interpretive qualitative study was conducted with 165 family caregivers of adults with CP across four Spanish regions (Andalusia, Aragon, Castile and Leon, and Galicia). Interviews explored meanings attributed to independent living, perceived feasibility, support needs, caregiving experiences, and well-being. Data were analyzed using inductive thematic analysis within a reflexive framework.

**Results:**

Four main themes were identified: (1) *Independence* was understood heterogeneously, ranging from rights-based conceptions centered on dignity, choice, and control to more restrictive views equating independence with functional self-sufficiency, often perceived as unattainable under high support needs; (2) *Supports* emerged as a decisive enabling condition, with families emphasizing the need for intensive, continuous, and coordinated assistance while reporting persistent barriers related to accessibility, bureaucracy, and service fragmentation; (3) *Family caregiving*, was described as a moral obligation involving substantial personal, social, and emotional costs. And (4) *Well-being* was framed as a central priority, encompassing health, safety, emotional stability, and social participation, while revealing tensions between protection and autonomy.

**Discussion:**

The findings suggest that independent living for adults with CP is constrained less by individual attitudes than by structural, organizational, and relational conditions. Families play a pivotal role in sustaining autonomy, often at considerable personal cost, which may in turn limit both caregiver well-being and the autonomy of the person with CP. Strengthening rights-based, personalized, and adequately resourced support systems, together with sustained training and emotional support for families, is essential to advance independent living and inclusive community participation for adults with CP.

## 1 Introduction

The independent living movement historically emerged from the advocacy of people with disabilities themselves and, in parallel, from their families. It represented a paradigm shift from a predominantly medical and care-based conception of disability to a social and rights-based approach (1, 2). Understanding disability from this perspective implies moving away from viewing the person as an object of care interventions and recognizing them as a subject of rights, with inherent dignity, autonomy, and the ability to participate fully in community life (3, 4). This framework was normatively consolidated with the Convention on the Rights of Persons with Disabilities (CRPD), which establishes fundamental principles such as self-determination, full participation and inclusion, equal opportunities, and accessibility (5).

Within this framework, Article 19 of the CRPD recognizes the right of all persons with disabilities to live independently and be included in the community, emphasizing freedom to choose where and with whom to live, as well as access to the supports necessary to make this possible (5). This is a cross-cutting right, as it underpins the exercise of other rights in community settings—such as education, employment, and social and political participation—within one’s chosen place of residence and with appropriate supports (6).

Nevertheless, the literature has consistently documented persistent misinterpretations of independent living. It is often reduced to “living alone” or “doing things without help,” an ableist reading that tends to exclude those who require extensive or continuous supports (7–9). Several authors warn that this reductionist definition legitimizes practices that, although nominally community-based, reproduce institutional logics, external control, and dependency (10–12). Consequently, independent living cannot be reduced to a mere residential change; rather, it must be analyzed as a dynamic and relational process shaped by support intensity, environmental accessibility, and the organization of care systems.

In the disability field, models of self-determination and quality of life have shown that the ability to exercise control over one’s own life depends more on real opportunities for choice, personalized supports, and accessible contexts than on individual functioning level (3, 13–16). From this perspective, independent living implies control over supports, not the absence of them. Independent living is not conceived as the absence of dependency, but as the capacity to exercise control and make meaningful decisions with appropriate supports (17–19).

Independent living has also been associated with significant improvements across key quality of life dimensions, such as emotional well-being, participation, interpersonal relationships, and self-determination (10, 20–26). However, these benefits do not emerge automatically. Even after transitions to community living, high levels of inactivity, social isolation, and limited meaningful interaction may persist when supports are ineffective, inconsistent, or insufficiently oriented toward active participation (7, 11, 13, 27–29). This evidence suggests that independent living depends not only on residential resources but also on broader cultural, organizational, and relational conditions, as well as on the centrality of the person in decision-making processes (11, 21, 30, 31), specifically for individuals with greater support needs.

This debate is particularly complex in the field of neurodevelopmental disorders and, specifically, cerebral palsy (CP). CP is the most common motor condition in childhood and is characterized by high heterogeneity in motor functioning, communication, cognition, sensory processing, and behavior (32, 33). Across the life course, many individuals with CP require intensive, continuous, and coordinated supports, which has historically limited access to independent living experiences in adulthood (34, 35). Despite advances in early intervention, rehabilitation, and assistive technology, a substantial gap remains between childhood services and available adult supports, particularly in terms of community participation and attainment of life goals (34, 36).

Although research on adult life in CP has traditionally been limited and focused on functional or health outcomes, recent studies have increasingly linked independent living from more community- and context-focused perspectives. The transition to independent living among adults with CP, including those with high support needs, involves not only the development of practical skills but also complex relational and emotional processes, such as renegotiating boundaries with caregivers and families, building an adult identity, exercising self-determination, and managing structural barriers (37, 38).

In this scenario, families play a central, enduring role. Caring for individuals with CP and other complex disabilities often entails assuming roles that exceed normative parenting functions, combining physical care, health coordination, support management, rights advocacy, and emotional accompaniment (39–41). This prolonged caregiving trajectory is associated with high levels of physical and emotional burden and has a significant impact on caregiver mental health and well-being.

Systematic reviews indicate a higher prevalence of depressive and anxiety symptoms among parents of people with CP, particularly in contexts of greater functional severity and more limited availability of formal supports (42, 43). Family perceptions of independent living should therefore not be interpreted in isolation as individual attitudes or beliefs, but rather as responses shaped to prolonged experiences of care, uncertainty, and sustained responsibility. Families may both facilitate independent living processes and experience tensions when protective practices translate into restrictions on decision-making or when expectations regarding capacity are constrained by deficit-oriented views (9, 26, 29, 44).

Alongside aspirations for community life, families experience concerns related to safety, well-being, and the long-term sustainability of care, particularly when support systems are perceived as insufficient, fragmented, or unstable (8, 45, 46). In CP, these concerns tend to intensify due to perceived vulnerability and increase as family caregivers age (47).

Although existing research has examined families and caregiving in CP and independent living in intellectual disability, relatively few studies have directly and systematically explored how families of people with CP conceptualize independent living, the conditions they deem necessary, and how they navigate tensions among self-determination, safety, well-being, and care sustainability. This gap is problematic, as it limits understanding of the effective realization of the right to independent living and constrains the development of viable and sustainable policies and services that do not implicitly shift structural responsibility onto households (10, 48, 49). Incorporating the family perspective does not displace the voice of the person; rather, it recognizes the relational and contextual nature of independence and contributes to the effective implementation of Article 19 of the CRPD.

Within this framework, the aim of this study was to explore family members’ perceptions of independent living for people with CP and the supports required to exercise this right, using a qualitative approach. Specifically, the goal is to analyze the meanings attributed to independent living, the perceived barriers and facilitators, and the implications for the design of supports aimed at promoting inclusion and quality of life for people with CP and their families.

## 2 Materials and methods

This study is grounded in interpretive phenomenology, an epistemological stance that understands knowledge as emerging from individuals’ lived experiences and their interpretation. From this perspective, reality is approached through meaning-making processes, and researchers are not neutral observers but engaged interpreters of participants’ accounts. Interpretive phenomenology seeks to explore how individuals make sense of their experiences and to illuminate both shared patterns and variations within those experiences, thereby identifying the underlying meanings of the phenomenon under study (50, 51). Data were analyzed using inductive thematic analysis as a flexible analytic strategy consistent with this interpretive framework (52).

### 2.1 Participants

A total of 165 family members of people with cerebral palsy (CP) who use services provided by organizations within the Spanish Confederation of Associations for the Care of People with Cerebral Palsy (ASPACE) participated in the study. Participants resided in four different Spanish regions: Andalusia, Aragon, Castile and Leon, and Galicia. Most participants were mothers of relatives with CP, with the remaining participants being siblings or fathers. Participants’ age was not systematically recorded, a limitation that should be considered when interpreting findings related to aging processes and caregiver burden.

Family members provided information about adults with CP aged 18 to 76 years (M = 41.5, SD = 14.0); 52.7% of the adults with CP were women. Functional mobility, manual ability, and communication were described using internationally recognized classification systems: the Gross Motor Function Classification System (GMFCS; 53), the Manual Ability Classification System (MACS; 54), and the Communication Function Classification System (CFCS; 55). The distribution across levels was as follows: GMFCS levels I–V (6.1%, 12.7%, 11.5%, 22.4%, 47.3%), MACS levels I–V (9.1%, 19.4%, 19.4%, 23.0%, 29.1%), and CFCS levels I–V (25.5%, 29.7%, 15.2%, 16.4%, 13.3%). Regarding intellectual disability, 6.0% of the individuals had mild disability, 11.5% moderate disability, 36.4% severe disability, and 46.1% profound disability. Concerning living arrangements, 53.9% lived in the family home, while 46.1% lived in residential settings.

Inclusion criteria were defined as being a family member of a person with a diagnosis of CP who uses ASPACE services and providing informed consent to participate. No exclusion criteria were established.

### 2.2 Instruments

A standardized open-ended interview format was used to ensure consistency across interviewers and regions while preserving participants’ opportunity to express their perspectives in their own words. The use of a common interview guide supported comparability across interviews and enhanced methodological transparency (50).

The interview guide addressed four main areas: (1) meanings and experiences associated with independent living (i.e., “What do you understand by independent living?”); (2) perceptions regarding the perceived feasibility of achieving independent living and the factors that facilitate or hinder it; (3) the supports available and required for independent living for people with CP and their families; and (4) the role of the family in promoting autonomy, perceived training needs, and the conditions considered necessary to foster a more independent and autonomous life for the family member with CP.

Prior to data collection, the interview guide was reviewed by a panel of experts and professionals in the field of disability and independent living, linked to the associative movement, with the aim of assessing the clarity, relevance, and accessibility of the language, as well as its suitability for families of individuals with diverse levels of support needs. Based on this review, minor wording adjustments were introduced to improve clarity and comprehension, without altering the substantive focus or structure of the guide.

### 2.3 Procedure

Data collection was carried out in collaboration with ASPACE-affiliated organizations and services across the participating autonomous communities. Professional teams within these organizations acted as initial mediators, informing families about the study and facilitating contact with those interested. Once participation was confirmed, interviews were scheduled.

Interviews were conducted by four professionals affiliated with participating ASPACE organizations, who acted as facilitators in the data collection process. Their involvement supported access to families and contributed to a climate of trust. To ensure consistency across interviews, all interviewers followed the same standardized interview guide. All interviews were transcribed verbatim by the four professionals who conducted them, following a standardized Word document template that included participant identification codes, the association they belonged to, each interview question, and space for participants’ responses. The transcriptions were reviewed by the research team to ensure accuracy prior to being imported into Atlas.ti software for analysis. In cases where interviews were conducted in person, by telephone, or through written responses due to logistical constraints, identical templates and procedures were applied to ensure consistency.

Interviews were conducted primarily by telephone, a strategy that helped maximize participation and reduce logistical, travel-related, and time constraints. In certain regions and specific cases, interviews were conducted in person (e.g., at the participant’s home when this option was more accessible) or using a mixed format that combined telephone and face-to-face encounters. In a limited number of cases, when distance or availability made synchronous interviews impractical, the interview guide was sent in writing for completion and return, while maintaining identical content and sequencing of questions.

Overall, participation levels were high, and most families engaged openly, often sharing their experiences in depth. In a small number of interviews, responses were more concise or interaction was less fluid; however, the pace and boundaries established by each participant were consistently respected.

The study was approved by the Ethics Committee of the University Institute for Community Integration, University of Salamanca (Ref. CEI-INICO/2023-01). Informed consent, confidentiality, and the right to withdraw at any time were guaranteed. Data were stored securely and in accordance with Spain’s Organic Law 3/2018 and the EU General Data Protection Regulation (EU) 2016/679, as well as with the principles of the Declaration of Helsinki and the regulations of the University of Salamanca.

### 2.4 Data analysis

Thematic analysis, recognized by Braun and Clarke (52) as an epistemologically flexible analytic approach compatible with multiple theoretical perspectives, was employed within an interpretive phenomenological orientation to explore and interpret participants’ lived experiences. Two researchers (the second and third authors) were actively involved throughout the analytic process. Prior to formal coding, they engaged in reflexive discussions to articulate and critically examine their assumptions and theoretical positions, acknowledging that interpretation is inevitably shaped by the researchers’ perspectives.

Each researcher independently read the interview transcripts to achieve immersion in the data and to generate inductive, data-driven codes. Through an iterative process of dialogue and comparison, codes were refined, grouped, and organized into broader thematic categories. For example, the codes ‘Neighbor support’ and ‘Volunteering’ were grouped under the theme ‘Community support,’ which, together with ‘Family support’ and ‘Social support,’ was further consolidated into a broader category labeled ‘Informal support’. This evolving set of codes enhanced analytic coherence and transparency throughout the process, while allowing the researchers to remain open to new insights emerging from participants’ experiences. All interviews were managed and coded using Atlas.ti software.

Consistent with the phenomenology approach, no inter-rater reliability coefficients were calculated. Instead, rigor was addressed through strategies commonly used in interpretive qualitative research, including analytical triangulation through ongoing researcher discussion, active consideration of divergent or contradictory cases, and explicit attention to reflexivity and methodological transparency, which together enhance credibility without reducing analytic depth (50, 56). Given the interpretive nature of the study, reflexivity was an integral component of the research process. The researchers involved have academic training and professional experience in disability studies and rights-based approaches, which inevitably informed their analytic sensibilities. To address this, interpretations were continuously discussed and contrasted, analytic positions were made explicit, and particular attention was paid to capturing the diversity and complexity of participants’ accounts, favoring a situated, transparent, and reflexive interpretation rather than claims of neutrality. For example, although the researchers identify with a rights-based perspective, which emphasizes that all individuals have the right to live independently, they deliberately coded data reflecting physical or intellectual requirements, even when these contradicted their own values. This demonstrates awareness of potential biases and illustrates how reflexivity was actively applied during the analytic process.

Finally, each of the four themes was developed and reported, illustrated with verbatim excerpts that reflect the diversity of family perspectives.

## 3 Results

The analysis identified four overarching themes: (1) *Independence*, (2) *Supports*, (3) *Family caregiver*, and (4) *Well-being of the person with CP*. Themes are summarized in **Table 1** and described below, supported by illustrative verbatim quotations presented in italics and quotation marks.

**Table 1:** Themes and subthemes identified in the analysis.

Theme	Subthemes
Independence	Rights and dignity; skills learning; housing type; economic conditions.
Supports	Intensity and continuity; physical and cognitive accessibility; sources of support (associative, informal, professional, technical); bureaucracy and coordination.
Family caregiver	Care as duty; relational bond; trade-offs and reconciliation; overload and aging; need for respite, training, and emotional support.
Well-being	Basic care and health; emotional well-being; behavior and psychological support; safety and overprotection; social participation.

### 3.1 Independence

Families expressed diverse and sometimes contrasting conceptions of independent living. For some, it was framed as a right linked to dignity, normalization, and the ability to choose, regardless of the level of support required. For others, however, independent living was primarily associated with functional self-sufficiency, leading them to view it as unfeasible when disability was perceived as “very severe”.

*“First, there is a legal mandate for this to be so, and fundamentally because, being a recognized right, it is my daughter’s wish and—just as with any child—we will try to make it a reality.”*

*“It all depends on the degree of disability. In my daughter’s case, how could she have an independent life! Everything has to be done for her. She would need a lot of continuous help.”*

In addition, several families emphasized the importance of learning and skills acquisition, often attributing a substantial role to individual effort and educational opportunities. Those who conceptualized independent living in terms of autonomy and decision-making generally considered it compatible with receiving support for task execution, although they highlighted the financial burden associated with high-intensity support arrangements.

*“That a person can take the reins of their life and make the decisions that affect them, relying on their own means and the supports necessary to do so when needed.”*

*“I don’t see it for people with a very severe disability or with limited financial resources.”*

Regarding housing, some participants explicitly linked independent living to leaving the family home, whether to live alone or in supported apartments. Less frequently, others suggested that independent living could also take place within residential settings, provided that sufficient control over daily routines and decisions was ensured.

*“To leave the family home just like their siblings did.”*

Finally, the economic dimension emerged as a decisive condition for achieving independence. Families underscored the substantial costs associated with securing adequate supports. Many pointed to the need for increased public benefits, while others highlighted persistent barriers to accessing and maintaining paid employment as a pathway to financial autonomy.

*“It is difficult to access independent living without financial backing or support from public institutions.”*

*“They would need paid employment … they take courses, do internships, and then they aren’t hired.”*

### 3.2 Supports

Supports were described as an essential element in enabling independent living, with families emphasizing the need for intensive, continuous, and reliable support to ensure meaningful social participation. Several participants questioned whether part-time support arrangements were sufficient for individuals with CP who have extensive or complex needs.

*“I don’t think they are sufficiently supported; part-time help isn’t enough. This group needs almost 24-hour support.”*

Persistent barriers to accessibility—both physical and cognitive—were identified as major constraints on mobility and community participation, reinforcing calls for universal design in urban planning and service provision.

*“It wasn’t until the elections came around that they started fixing the crosswalks and curb cuts. We’ve been living here for 21 years, and in all that time we’ve had a lot of trouble.”*

*“Cognitive accessibility in urban buses that would facilitate better understanding of routes…”*

With regard to sources of support, the associative movement was highlighted as a central point of reference and trust, although participants also reported difficulties related to inter-institutional coordination and limited access to clear, consistent information about available services.

*“From [organization], we have a lot of backing and trust.”*

Professional support was also highly valued, particularly when it was personalized and delivered by well-trained, stable staff. Adequate staffing ratios were considered critical for ensuring continuity, safety, and service quality.

*“I’m very grateful for the support and care … although at night I see there is only one person on duty.”*

Informal support from other family members and community actors was also emphasized as an indispensable complement to formal supports for daily care and social integration.

*“Yes, I do feel social support. We were among the first to live in the neighborhood; people know us, and I like that they understand my daughter’s situation—we have to normalize it.”*

Participants further mentioned technical supports (e.g., home automation and assistive technologies) and psychological support as important resources to improve functioning and overall well-being.

*“Modern technology … assistive tools … are making it easier to live independently.”*

Finally, families expressed strong criticism of political and administrative support, citing bureaucracy, lengthy delays, and regulatory gaps that hinder timely access to essential resources.

*“When you apply for assistance, it can take years to arrive.”*

Overall, these findings underscore that both formal and informal supports must be understood within a comprehensive and coordinated framework, addressing not only the intensity and continuity of assistance but also structural barriers and inter-system fragmentation.

### 3.3 Family caregiver

The role of the family caregiver emerged as structural and indispensable in sustaining independent living for people with CP. Many participants described caregiving as a responsibility assumed “naturally” within the family, experienced primarily as a moral obligation rather than a freely chosen role.

*“Your child is yours, and you have to be the one to raise him.”*

The caregiver–care recipient relationship was portrayed as a complex bond characterized by deep trust and emotional closeness, alongside mutual dependence. While this dynamic was often described as protective, families also acknowledged that it could constrain autonomy on both sides.

*“It can also help reduce tension in the family–caregiver relationship, allowing greater autonomy for the person.”*

Daily caregiving commitments involved substantial personal, professional, and social trade-offs. Participants described reduced leisure opportunities, strained interpersonal relationships, and difficulties maintaining an active social life, often leading to isolation and challenges in work–life balance.

*“I had to reduce my working hours to care for my daughter.”*

*“Periodic breaks … a respite to regain strength and keep up with this pace of life and sacrifice.”*

Physical and emotional overload intensified with caregiver aging. Increasing support needs, combined with caregivers’ own health decline, generated sustained strain that frequently manifested as chronic fatigue, stress, and symptoms of caregiver burden.

*“There comes a time when you get tired and worn out psychologically.”*

*“We are getting older and older, and although we are still capable, there are some situations in which it is more difficult for us to help our daughter.”*

Another recurring element was the lack of specific training to address physical, health-related, or behavioral demands, compounded by difficulties accessing training opportunities due to caregiving responsibilities. Many caregivers reported feeling insufficiently prepared, which undermined confidence and sometimes compromised perceived care quality. Consequently, access to ongoing, practical training was identified as a priority need.

*“Courses that we cannot always attend because of caregiving responsibilities.”*

*“I would like training … in physiotherapy, nursing, and psychology.”*

Participants also emphasized the importance of social recognition and emotional support as protective factors. Opportunities to share experiences with other caregivers—such as peer support groups—were viewed as valuable mechanisms for reducing isolation, exchanging knowledge, and strengthening coping strategies.

*“It’s important to have a* sp*ace to share experiences and receive emotional and psychological support.”*

*“And thanks to sharing experiences with other parents.”*

### 3.4 Well-being of the person with cerebral palsy

Well-being emerged as the central organizing priority in family discourse, encompassing health, emotional stability, safety, and social participation.

Families first emphasized the importance of maintaining basic care—adequate nutrition, rigorous hygiene, and continuous medical follow-up—not only to prevent physical deterioration but also to sustain long-term quality of life.

*“Specialized medical care, physical and occupational therapy.”*

*“Caregivers must take on multiple responsibilities such as medical care, medication administration, mobility, feeding, or personal care…”*

Emotional well-being was also highlighted as a key concern. Families identified needs related to managing behavioral challenges and accessing psychological support from early stages, noting that appropriate intervention facilitated coexistence and reduced household tension. The availability of specialized professional support was seen as essential for both family functioning and personal development.

*“Facing difficult situations, managing behaviors and mood—both theirs and ours.”*

*“Psychological help … necessary from the moment of diagnosis.”*

*“Being able to do the small things that bring satisfaction and allow them to feel fulfilled.”*

Families further noted that acceptance of disability by the person with CP played a decisive role in life satisfaction. Positive adjustment was associated with higher self-esteem and greater motivation to engage in meaningful activities, benefiting both the individual and overall family balance.

*“Teaching them to live with their disability.”*

Safety concerns emerged as another salient dimension of well-being. Fears related to accidents, abuse, or risky situations often led to overprotective practices. Although vigilance was intended to safeguard the person, participants recognized that excessive protection could become a barrier to autonomy, limiting opportunities for learning and self-efficacy.

*“I’ve become afraid … we used to leave him alone for longer periods of time.”*

*“I admit I didn’t let her go out alone because I was very afraid that something would happen to her. Sometimes it’s the mothers’ fault.”*

Finally, social participation was described as essential for community inclusion and the development of support networks. Inclusive leisure activities and opportunities to build interpersonal relationships were valued as key contributors to daily satisfaction, a sense of belonging, and family resilience.

*“I think she would need a friend so she could go out more—like any 27-year-old person.”*

*“Freedom of movement … going for a walk, going to a concert.”*

## 4 Discussion

The present study explores families’ perceptions of independent living for people with cerebral palsy (CP), revealing a complex and heterogeneous understanding shaped by structural, contextual, and emotional factors. Findings indicate that independent living is experienced as a dynamic and ongoing process in which a rights-based framework, the availability and quality of supports, and the well-being of both individuals with CP and their families are deeply intertwined.

First, the results show the coexistence of divergent conceptions of independent living. Some families understand it as an inherent right—consistent with Article 19 of the CRPD—linked to dignity, normalization, and the possibility of choice, whereas others equate it with the functional ability to perform activities of daily living without assistance. The latter interpretation leads, in some cases, to viewing independent living as unfeasible when disability is perceived as highly demanding or as entailing extensive support needs.

These findings are consistent with documented tensions between rights-based normative models and ableist conceptions that equate independence with functional autonomy (9, 57). From contemporary self-determination and support frameworks, independence is not defined by the absence of supports but by control over meaningful life decisions and the ability to direct one’s own life with the necessary supports (14, 16, 18). However, the present results indicate that this theoretical distinction does not always translate into family representations, especially when required supports are continuous, high-intensity, and perceived as insufficient, a pattern also identified in previous studies (44–46).

In this regard, the perception that independent living is unfeasible appears to derive not only from restrictive attitudes but also from a pragmatic and experience-based appraisal of the material, economic, and organizational conditions available in families’ environments. Within this context, the findings highlight a tension between the normative recognition of independent living as a right and the structural barriers that hinder its implementation. However, independent living cannot be reduced to an ideal contingent upon the level of support required. Rather, it refers to the extent to which individuals can exercise participation, choice, and control over their own lives. From this perspective, its realization is shaped less by the severity of disability itself than by the availability and adequacy of supports that enable individuals to pursue their own goals, interests, and preferences.

The results reinforce the evidence of a persistent gap between rights-based discourse and the conditions required for effective implementation, widely noted in research on independent living and community supports (10, 12, 58). The results also show that some families recognize alternative housing arrangements—such as supported apartments or certain small residential settings—as compatible with independent living, provided that meaningful control over daily life is preserved.

Second, supports are positioned as the central axis enabling independent living. Independent living is constructed within contexts of interdependence and through supports that facilitate decision-making and control over significant aspects of daily life (59). Families describe the need for high-intensity, continuous, and high-quality supports, explicitly questioning the adequacy of partial or fragmented interventions. This perception is consistent with evidence indicating that intermittent support models are insufficient for people with physical, communication, and cognitive disabilities and may generate risk, exclusion, or unwanted dependency (13, 21, 22, 29).

Persistent physical and cognitive accessibility barriers emerge as obstacles that limit community participation, reinforcing that independent living depends not only on personal supports but also on accessible environments and universal design. Despite international obligations regarding accessibility (5, 6), Spain continues to exhibit serious deficits, particularly in housing, transport, leisure, and culture (60, 61). Efforts to promote cognitive accessibility remain limited and lack a common strategy, highlighting the gap between regulations and effective implementation (62). The findings also underscore the central role of the associative movement as a source of support, information, and trust, in contrast to public systems perceived as bureaucratic, inflexible, and poorly coordinated.

The combination of formal and informal supports appears both indispensable for sustaining independent living and indicative of the externalization of structural responsibilities onto families and communities. From a public policy perspective, this underscores the need for comprehensive, adequately funded support systems that do not implicitly rely on the unlimited availability of informal networks (6, 31).

Third, the role of the family caregiver is positioned as a structural element for sustaining independent living, albeit at a high personal cost. Care is described as a responsibility assumed “naturally,” more closely tied to moral duty than to an explicit choice. Testimonies reflect an ambivalent relationship between care and autonomy: while the bond of trust and protection is perceived as essential, it is also acknowledged that this dynamic can restrict the autonomy of both the person with a disability and the caregiver. This tension is consistent with studies showing that intensive care can generate mutual dependency in the absence of adequate external supports (38–41, 49, 59).

Physical and emotional overload—exacerbated by caregiver aging and growing support needs—emerges as one of the most consistent findings. The literature has extensively documented the impact of prolonged caregiving on mental health, labor participation, and family quality of life (42, 43). The lack of specific training and respite supports further undermines families’ coping capacity and contributes to insecurity in caregiving practices.

Finally, the well-being of the person with CP is positioned as a central priority in family discourse, articulated around health, emotional stability, safety, and social participation. This conception aligns with multidimensional quality-of-life models integrating physical, emotional, and social domains (3, 14). Quality of life is also closely linked to the provision of supports, contributing to decision-making for full participation (63, 64).

Although independent living and quality of life may seem closely related in family narratives, it is necessary to clarify that they are not equivalent concepts. Independent living emphasizes the right to autonomy, personal choice, and control over one’s own life, as articulated in Article 19 of the CRPD (5). In contrast, quality of life refers to a broader multidimensional framework that encompasses multiple domains of well-being (3). While both share a rights-based orientation and relate to self-determination, participation, and social inclusion, independent living cannot be reduced to quality of life, nor does quality of life fully reflect the structural dimensions inherent in the independent living framework. In this sense, it should not be assumed that independent living automatically leads to improved quality of life outcomes, but rather that it establishes the structural conditions under which such outcomes may be enhanced if effective, consistent, and participation-oriented supports are in place (20, 21).

Social participation emerges as a key component of well-being, valued both for its impact on the quality of life of the person with CP and for its protective effect on the family. Fear of accidents, abuse, or risky situations appears as a factor fueling overprotective practices, which families themselves recognize as potential barriers to independence. This finding is consistent with research showing that risk perceptions can limit learning and participation opportunities, particularly in contexts where community supports are insufficient or unstable (20, 25, 27). The demand for recognition, emotional support, and peer exchange spaces underscores the importance of interventions directed not only at the person with a disability but at the family system as a whole.

### 4.1 Study limitations

This study has several limitations that should be considered when interpreting the results.

First, the analysis focuses exclusively on family perspectives, implying a necessarily partial view of independent living. While this focus aligns with the study aim and enables deeper insight into families’ structural role in organizing and sustaining supports, the findings do not replace or claim to represent the voices of people with CP themselves. Rather, they capture one relational position within a broader support ecology. Future research should systematically incorporate both perspectives to explore convergences and divergences in the conceptualization and experience of independent living.

Second, the findings are situated within a specific sociopolitical and service context, shaped by particular institutional frameworks, patterns of support provision, and families’ connections with associative organizations. This contextual embeddedness may influence access to resources, information, and support trajectories, potentially limiting the transferability of the results to settings with different welfare regimes or service configurations. However, from a qualitative standpoint, the aim is not statistical generalization but the generation of analytically transferable knowledge relevant to contexts with comparable characteristics.

Additionally, the use of standardized open-ended interviews and collaboration with associative organizations represents a methodological choice that prioritizes accessibility, comparability, and broad participation. While this approach facilitated the inclusion of a large and diverse group of families across regions, it may have shaped the depth or framing of some narratives. Future studies could complement this approach with in-depth or longitudinal qualitative methods to further explore individual trajectories and evolving meanings of independent living over time.

Finally, although the study encompasses a wide range of family experiences, it was not designed to systematically analyze differences related to level of support needs, age of the person with CP, or type of residential arrangement. These dimensions emerged as salient in family narratives and constitute important avenues for future research aimed at deepening understanding of independent living across diverse life trajectories.

### 4.2 Implications and future research

The study’s findings have relevant implications for research, practice, and policy. First, they reinforce the need to conceptualize independent living through relational and contextual lenses that acknowledge diverse life trajectories and move beyond normative models grounded solely in functional self-sufficiency. This perspective is consistent with a social and rights-based understanding of disability as a relational phenomenon shaped by the interaction between individual conditions and environmental contexts, rather than as an inherent individual characteristic traditionally proposed by the medical model. Second, they underscore the urgency of developing comprehensive, flexible, and adequately funded support systems capable of reducing overreliance on informal networks and alleviating sustained family burden.

From a practice standpoint, the results contribute to a more precise understanding of what family-centered interventions entail in the domain of independent living. Families do not request generic information alone, but rather concrete, applied training to address the physical, health-related, and emotional demands of daily care, alongside sustained supports that allow them to accompany autonomy processes without compromising their own well-being. Independent living is perceived as a prolonged and emotionally demanding process, strongly conditioned by caregiver aging and uncertainty about the future. This highlights the need for stable, long-term accompaniment strategies rather than interventions limited to isolated transition or emancipation milestones. In this context, assistive technologies and universal design are valued for their potential to expand autonomy and participation, but only when integrated within sufficiently coordinated, accessible, and person-centered support networks.

Several priority lines for future research and intervention emerge from the findings, particularly from a family perspective. First, there is a need to design and evaluate flexible training programs for family caregivers that are responsive to the real demands of intensive and long-term care. Future studies should examine accessible training formats—such as modular, distance-based, or in-home approaches—and assess their impact not only on caregiver competencies, but also on the autonomy, safety, and well-being of the person with a disability.

Second, the findings highlight the importance of longitudinal psychological support for families throughout transitions to independent living. Psychological support is described in family accounts as a structural and ongoing need rather than as a time-limited resource. This points to the need to evaluate continuous psychological accompaniment models that support emotional regulation, address fear of risk, and mitigate uncertainty about the future, thereby facilitating a more sustainable balance between care, well-being, and the promotion of self-determination.

Lastly, the findings underscore the importance of examining perceptions of safety and risk as mediating variables in independent living processes. Fear of accidents, abuse, or vulnerability emerges as a central factor in family decision-making and is sometimes invoked to justify overprotective practices that restrict opportunities for independence. Future research should explore strategies for creating environments that are both safe and enabling, without constraining self-determination. Addressing this tension is essential for advancing independent living models that balance protection, well-being, and rights.

## Data Availability

The raw data supporting the conclusions of this article will be made available by the authors, without undue reservation.
